# Unique relationship between osteophyte and femoral-tibia component size mismatch in determining polyethylene wear in primary total knee arthroplasty: a case report

**DOI:** 10.1186/1752-1947-3-59

**Published:** 2009-02-10

**Authors:** Manjunath Ramappa, Andrew Port

**Affiliations:** 1 James Cook University Hospital, Middlesbrough, UK

## Abstract

**Introduction:**

Knee pain is a complex problem that can occur after total knee arthroplasty. One cause of knee pain may be due to a retained osteophyte, but it is not clear if the retained osteophyte is sufficient explanation of the pain, as not all patients with retained osteophytes are symptomatic. In fact, the literature shows that excised osteophytes can also recur over a period of time, without any symptoms. Therefore a retained osteophyte alone is probably not sufficient to cause symptoms.

**Case presentation:**

We present a case of intermittent medial knee pain occurring post-primary total knee arthroplasty, in a patient who underwent several investigations over a period of 5 years. Radiographs showed an osteophyte in the postero-medial femur along with slight tibial component overhang which was normal for that knee implant design. The symptoms eventually settled with excision of only the osteophyte, without altering the tibial component.

**Conclusion:**

A retained osteophyte alone, or tibial component overhang alone, did not seem to cause significant symptoms in our patient whose symptoms completely settled with excision of the osteophyte alone, without changing the tibial component. Therefore, it seems that the combination of retained osteophyte and tibial component overhang (tibia-femoral component size mismatch) are detrimental and therefore best avoided. This report also emphasises the importance of meticulous osteophyte excision and avoiding tibial component overhang during knee arthroplasty.

## Introduction

This case report discusses knee-implant designs which have natural femoral-tibial component mismatch with tibial component overhang, and their unique association with the surrounding soft tissues, especially retained osteophytes. To our knowledge, this relationship has never been described before.

Total knee arthroplasty (TKA) is an effective means of providing pain relief for patients with arthritic knees. There appears to be rapid and substantial improvement in the patient's pain, functional status and overall health-related quality of life in about 90% of cases. However, in a few patients, pain persists even after arthroplasty. Successful treatment of this pain depends on the cause [1]. If the cause is a rarity, it becomes a diagnostic as well as therapeutic challenge.

Pain can have a mechanical origin, when it is caused by loosening or component failure, or it can be continous when associated with infection. Delayed-onset pain is usually associated with infection or inflammation (synovitis, tendonitis, wear process) [2].

Variables that affect the wear of a polyethylene bearing in vivo include the following: wear resistance of the materials as well as the loads, lubrication, sliding distance, motion pattern, specifics of the design and manufacturing of the polyethylene component, implantation techniques, type of wear and amount and type of use of the joint.

We know that component-size mismatch can contribute to instability after TKA [3]. The component-size mismatch, however, can be normal for some knee implant designs. Also, the mismatch can expose more surface area of that non-articulating polyethylene to surrounding tissues.

## Case presentation

**Figure 1:**
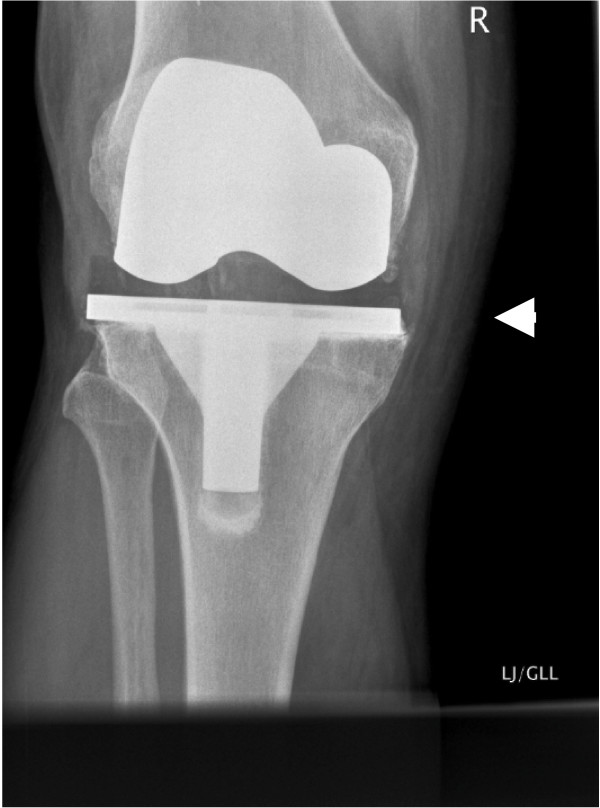
**Initial post-total knee arthroplastyradiograph, showing medial femoral osteophyte (arrow) and tibial component overhang compared to the femoral component**.

**Figure 2:**
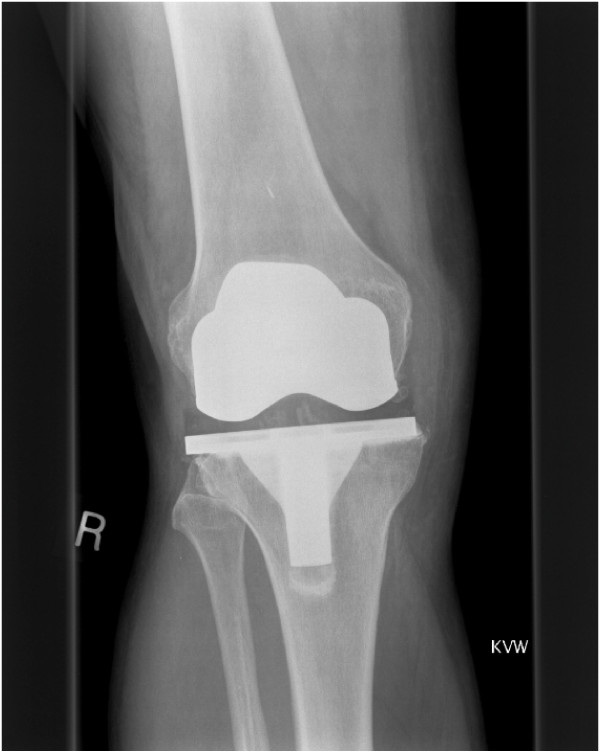
**Radiograph at 5 years post-total knee arthroplasty, with medial femoral osteophyte (arrow) and tibial component overhang compared to femoral component**.

**Figure 3:**
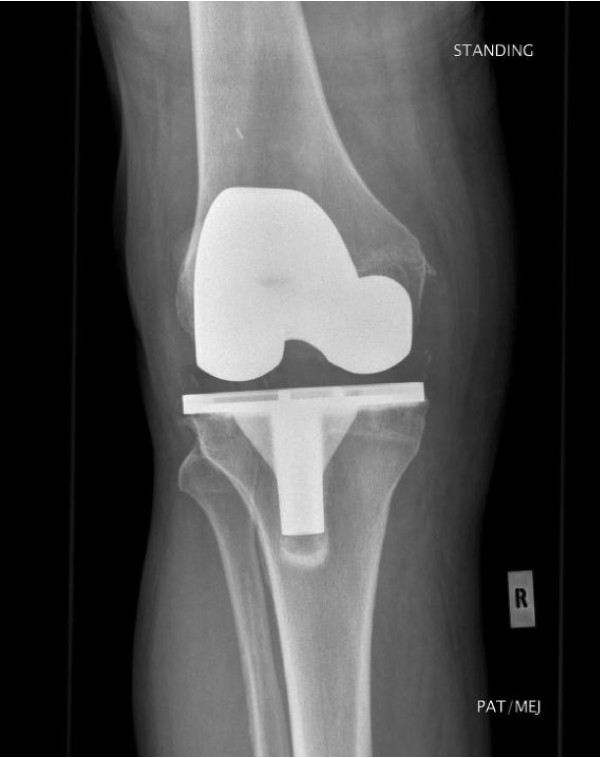
**Radiograph post-medial femoral osteophyte excision (arrow)**. Tibial and femoral components are unchanged.

A 64-year-old man had undergone right TKA for osteoarthritis, with a cruciate-retaining PFC knee system: size 5 femur and 5 tibia with a 10 mm posterior lipped tibial insert. The initial postoperative period was uneventful with 0 to 100 degrees of knee flexion. The X-ray (Figure 1) showed good knee alignment with a slight tibial overhang. An untrimmed osteophyte was identified at the postero-medial femoral condyle. In the first follow-up at 6 weeks, the patient complained of minimal pain and swelling at the anteromedial aspect of the knee. His symptoms progressed and, at 6 months, the patient underwent an arthroscopic exploration with washout and samples were sent for culture & sensitivity. All samples were negative for any microorganisms. C-reactive protein, white-cell count and erythrocyte sedimentation rate remained stable and the pain appeared to settle. At 1-year follow up, the patient had some medial knee pain which was controllable. At this stage, he seemed pleased with the outcome of the surgery. At 3 years, he presented at the clinic again due to recurrence of medial knee pain. X-rays showed no changes. Technetium 99 m diphosphonate bone scintigraphy showed increased uptake on the delayed phase mainly in the medial femoral and tibial condyle, which was inconclusive. Inflammatory markers were again stable. The pain disappeared shortly after the scan. The pain recurred once again 5 years after surgery and examination revealed a tender point at the medial joint line with a palpable lump and good range of flexion. No changes were observed on a repeat X-ray (Figure 2). At this stage, the medial joint line was explored which showed a small osteophyte at the postero-medial border of the femur, causing a localised polyethylene rim wear (non-articulating part) and localised medial synovial reaction. The osteophyte was excised. Tibial and femoral components were stable and hence not revised. At 2 years post-osteophyte excision (Figure 3), the patient was pain-free and asymptomatic. Throughout this period, the patient had good knee alignment with 0 to 100 degrees of flexion.

## Discussion

Intermittent knee pain following arthroplasty poses a significant therapeutic challenge. Arthroscopy has a limited role post-TKA [4]. The clinical triad of effusion, pain and progressive change in the alignment of the knee which is characteristic of accelerated polyethylene wear [5] is not always seen. Some causes of pain reported in the literature after total knee replacement include remnant soft tissues [6,7], polyethylene wear, low-grade infection, loosening, malalignment and over-stuffing. The intermittent exacerbation of symptoms is secondary to intermittent synovitis [8]. A conservative approach and reassurance has had success in the management of undiagnosed knee pain in the past [1].

'Wear' is the removal of material as a result of the relative motion between two opposing surfaces under load. Wear particles thus produced activate macrophages, which in turn release substances resulting in loosening and osteolysis. In a complex mechanical-biological system such as total knee replacement, there can be many types of wear. Polyethylene thinning, although commonly seen, is a complex type of wear.

Persistent osteophytes have been reported to cause problems occasionally [9,10]. Meticulous resection of the osteophyte is an important technique to prevent post-operative discomfort in the knee. Small osteophytes can be easily missed, especially in posterior compartments. These osteophytes can cause asymmetrical abrasive wear ('Mode-2 wear' [3]) of a non-articulating polyethylene surface. Asymmetrical wear of a polyethylene bearing can alter the mechanical axis of the knee and thereby increase the rate of wear in that compartment because of the increased load [8,11,12].

Friction is the resistance to movement between two surfaces in contact. Frictional torque is the force created as a result of the friction of bearing. In Mode-2 wear, significant frictional torque can accelerate the wear process.

Osteophytes can interact with the polyethylene and surrounding soft tissues to cause a synovial reaction and wear. If left untreated, osteophytes are known to enlarge in size. Recurrent osteophytes post-excision are not uncommon [10]. Retained osteophytes have been known to cause problems but may also be asymptomatic. Therefore, there has to be a further contributing factor for this synovial reaction and wear caused by osteophytes. Some knee implant designs have slight tibial overhang as compared with the femoral component of same size [13], exposing more area of the non-articulating polyethylene surface to surrounding tissues (Figure 1 to Figure 3). Consequently, there is an increased possibility of interaction between the non-articulating polyethylene and the surrounding tissues, including any persistent osteophytes. This, in turn, accelerates abrasive polyethylene wear and intermittent synovitis [14]. Therefore, retained osteophytes in combination with a large surface area of non-articulating polyethylene (as in our case, with tibial component overhang) can cause this type of synovial reaction and wear. In our patient, removal of the osteophyte, without changing the tibial implant, was sufficient to clear his symptoms. Therefore, neither of these two factors in isolation caused detrimental effects, but it is their combination which created this situation. Therefore, treatment should be directed at addressing either or both of these issues.

## Conclusion

This report provides evidence that the combination of osteophyte and tibial component overhang can be detrimental after TKA. We further show that this can be resolved by addressing either or both of the detrimental factors. This report emphasises the importance of meticulous osteophyte excision and avoiding tibial component overhang during TKA.

## Consent

Written informed consent was obtained from the patient for publication of this case report and accompanying images. A copy of the written consent is available for review by the Editor-in-Chief of this journal.

## Competing interests

The authors declare that they have no competing interests.

## Authors' contributions

MR and AP 1) both made substantial contributions to conception and design, acquisition and interpretation of the data; 2) were both involved in drafting the manuscript or in revising it critically for important intellectual content; and 3) have both given final approval of the version to be published.
